# COVID-19: long-term respiratory consequences

**DOI:** 10.1590/1516-3180.2021.139526052021

**Published:** 2021-08-09

**Authors:** Carlos Toufen, Paulo Manuel Pêgo-Fernandes

**Affiliations:** I MD, PhD. Attending Physician, Division of Pulmonology, Instituto do Coracao, Hospital das Clinicas HCFMUSP, Faculdade de Medicina, Universidade de Sao Paulo, Sao Paulo, SP, BR; II MD, PhD. Full Professor, Thoracic Surgery Program, Instituto do Coracao, Hospital das Clinicas HCFMUSP, Faculdade de Medicina, Universidade de Sao Paulo, Sao Paulo, SP, BR; Director, Scientific Department, Associação Paulista de Medicina, São Paulo (SP), Brazil

Coronavirus disease-19 (COVID-19) was first documented in China in late 2019 and was declared a pandemic in March 2020. It has produced death and dysfunction around the world. In Brazil, as of May 20, 2021, there had been more than 15 million confirmed cases of COVID-19 and more than 400,000 deaths. Although COVID-19 was initially identified as a pneumonia-like illness, its pathophysiology in severe cases can include destruction of lung epithelial cells, thrombosis, hypercoagulation and vascular leakage. These events lead to acute respiratory distress syndrome (ARDS).

After one year of the pandemic, researchers are looking beyond the acute illness. Increased attention is being given, with considerable debate, to persistent symptoms and complications that have the potential to impose an appreciable burden of chronic respiratory symptoms or fibrotic disease on recovered individuals. No consensus has yet been reached regarding the terminology and clinical definition of the long-term consequences of COVID-19, but understanding of the issues is increasing rapidly.

The terms post-COVID syndrome or long COVID have been used in the literature, with the suggestion that this condition can be classified as subacute or ongoing symptomatic COVID-19 (symptoms and abnormalities present 4-12 weeks beyond acute COVID-19) and chronic or post-COVID-19 syndrome (symptoms and abnormalities persisting or present beyond 12 weeks after the onset of acute COVID-19 and not attributable to alternative diagnoses).^1^ In initiatives implemented to quantify and evaluate sequels, it has been estimated that around one in five people who tested positive for COVID-19 had symptoms that lasted for five weeks or longer, and one in ten people had symptoms that lasted for 12 weeks or longer.^1^ The World Health Organization (WHO) has recommended that patients who have COVID-19 (both confirmed and suspected) should have access to follow-up care if they have persistent, new or changing symptoms.^2^

Respiratory dysfunction has been described as one of the most prevalent symptoms in long COVID. In one study, dyspnea and chest pain were found at high proportions after two months among previously hospitalized patients.^3^ One third of the patients who required noninvasive ventilation or mechanical ventilation still presented dyspnea after six months. Even individuals who were not hospitalized were at greater risk of dyspnea during the first six months after acquiring COVID-19 than were controls who had not had COVID-19. Dyspnea was also found to be more frequent among patients hospitalized for COVID-19 than among those hospitalized due to influenza.^4^

Further respiratory symptoms have been seen to occur frequently in long COVID. Chest pain was present in a quarter of the patients discharged from hospital two months after COVID-19. At six months, the risk of chest pain, compared with patients without COVID-19, was higher. Dry cough, one of the most common symptoms of COVID-19, along with fever, anosmia and ageusia, can persist for weeks or months after COVID-19. Long COVID-19 patients have also presented evidence of high burden of incident use of bronchodilators, antitussives, expectorants, anti-asthmatics and glucocorticoids.^4^

Hypoxemia and exertional desaturation are associated with dyspnea and might represent pulmonary impairment in long COVID. The National Institute for Health and Care Excellence (NICE) guidelines suggest that all patients should be evaluated using an exercise tolerance test to record their levels of breathlessness, heart rate and O_2_ saturation.^1^ A one-minute sit-to-stand test (1STST) is currently used to evaluate long COVID-19 patients.

At six months, it was found that survivors presented greater risk of hypoxemia than did controls with other diagnoses. COVID-19 survivors after hospitalization were at threefold greater risk of hypoxemia than non-hospitalized COVID-19 survivors, and survivors from intensive care unit (ICU) admission presented sevenfold greater risk of this than did other COVID survivors. Comparison of patients who were hospitalized with COVID and those who were hospitalized due to seasonal influenza also showed that the risk of hypoxemia was higher among COVID patients.^4^

Functionally, reduced forced vital capacity (FVC), total lung capacity (TLC) and diffusing capacity (DLCO) have been found in patients with long COVID, but no airway obstruction. Lung function assessment at six months after COVID-19 showed that a considerable proportion of the participants had pulmonary diffusion abnormalities.^5^ At 12 months, residual abnormalities of pulmonary function were observed in a third of the patients, and the most common finding was a reduction in DLCO. DLCO was also independently correlated with greater severity of the initial disease.^6^

The higher forced expiration volume in first second (FEV1)/FVC ratio in the severe/critical subgroup suggested that it presented restrictive physiology. There was no difference between critical and non-critical patients in terms of respiratory muscle dysfunction strength, which suggested that a lung parenchymal rather than a respiratory muscle dysfunction was present.^7^

A negative correlation between the duration of mechanical ventilation and pulmonary function at a four-month follow-up has been demonstrated.^7^ This may have been due to prolonged or more severe disease after very severe COVID-19 or may have been related to ventilator-induced lung injury, which is a well-described challenge post-ARDS and can reduce pulmonary function after recovery.^8^

Respiratory viral infection might potentially induce distinct fibroblast activation in the convalescence phase.^5^ Lung dysfunction has been correlated with pulmonary interstitial change seen on computed tomography (ground-glass opacifications and reticulations/parenchymal bands). A mosaic attenuation pattern (abnormally hypodense areas alternating with normal or abnormally hyperdense areas) and air trapping have also been observed and may indicate small-airway disease. The association of mosaic attenuation pattern and impaired DLCO can cause ventilation-perfusion mismatch, thus contributing to reduced physical performance and hypoxemia. Potential development of progressive interstitial lung disease might be triggered by COVID-19, drug-induced lung injury or progression from pre-existing interstitial lung abnormalities.

Long COVID patients also present higher risk of thromboembolic diseases, and pulmonary emboli have been highlighted as a differential for dyspnea. At six months, survivors of COVID-19 were found to present greater risk of thromboembolism than controls with other diagnosis. After six months, COVID-19 survivors who had been hospitalized were at sevenfold greater risk of thromboembolism than were non-hospitalized COVID-19 survivors; and survivors of COVID-19 intensive care unit admission were at 18-fold greater risk than all other survivors of COVID. Comparison of patients who were hospitalized due to COVID with those hospitalized due to seasonal influenza showed that the risk of venous thromboembolism (VTE) was about twice as high after six months.^4^

Since the impact of COVID-19 varies from full recovery to severe and persistent debilitating symptoms, patients who remain symptomatic after four weeks should be considered for an initial evaluation to identify and treat disorders, with medication reconciliation and doctor referral if needed. The objectives in managing long-COVID are to support patients in the ambulatory setting, avoid the additional burden of predicted readmissions, understand the trajectory of each patient and identify therapeutic opportunities. Persistent respiratory symptoms should be evaluated to exclude diagnoses such as organized pneumonia, pulmonary embolism, heart disease, lung fibrosis and neuromuscular weakness.

No conclusive evidence for routine use of corticosteroids and anticoagulation in cases of long-COVID has been found. Therefore, currently, such use should be individualized based on functional and radiological findings, degree of symptoms and risk/benefit analysis. Cough and chest pain could be managed through administration of opioid derivatives. Gabapentin and pregabalin, which are neuromodulators, could be considered for treating post-COVID syndrome, although they have the potential to worsen any cognitive dysfunction. Antimuscarinic drugs, such as tiotropium, could be used to control COVID-19 cough, because these can decrease cough sensitivity in cases of acute viral upper respiratory tract infection.^9^

The importance of multidisciplinary rehabilitation has been acknowledged for patient management post-COVID. Rehabilitation programs should be adjusted to the needs of the patient and details are still required regarding who would benefit from rehabilitation, and what kind of rehabilitation this should be. It is also necessary to verify that sufficient capacity is available within community rehabilitation services to assist in cases of long COVID.^10^

Studies involving long COVID-19 are dynamic and are being published quickly, thereby updating comprehension of the biological basis of symptoms and the therapeutic opportunities for recovery and rehabilitation. It will be crucial to offer individualized, evidence-based care for these patients.
